# Exploring Salivary Biomarkers in Pediatric Obesity: A Scoping Review

**DOI:** 10.3390/ijms26125789

**Published:** 2025-06-17

**Authors:** Fernanda Maria Sabella, Renata Thomaz Katzenelson, Fabíola Galbiatti de Carvalho, Cristiane Duque, Michelle Darrieux, Fernando Augusto Lima Marson, Thaís Manzano Parisotto

**Affiliations:** 1 Laboratory of Clinical and Molecular Microbiology, Universidade São Francisco (USF), Bragança Paulista 12916-900, São Paulo, Brazil; fernanda.sabella@usf.edu.br (F.M.S.); renata.katzenelson@usf.edu.br (R.T.K.); michelle.bertoncini@usf.edu.br (M.D.); fernando.marson@usf.edu.br (F.A.L.M.); 2 Department of Pediatric Dentistry, School of Dentistry, Federal University of Uberlândia (UFU), Uberlândia 38400-902, Minas Gerais, Brazil; fabiola.carlo@ufu.br; 3 Centre for Interdisciplinary Research in Health (CIIS), Faculty of Dental Medicine, Universidade Católica Portuguesa (UCP), 3504-505 Viseu, Portugal; cduque@ucp.pt

**Keywords:** saliva, biomarkers, pediatric obesity

## Abstract

Childhood obesity and overweight are linked to subclinical inflammatory conditions. The present manuscript aimed to undertake a scoping review exploring the relationship between childhood obesity and salivary biomarkers to answer the following question: “Are salivary biomarkers trustful factors/indicators for childhood obesity?” The main search terms used were: “obesity and salivary biomarkers and children” (Pubmed, Scielo, Scopus, Embase databases: 1999–2025). Assessed articles were carefully classified according to a predetermined criterion (Newcastle–Ottawa Scale), and the Preferred Reporting Items for Systematic Reviews and Meta-Analyses (PRISMA) were considered. Papers involving children >13 years, duplicates/triplicates, literature reviews, and non-related to the question addressed were excluded. More than 30 salivary biomarkers were assessed in the thirteen studies appraised. Three studies were rated as having a high level of evidence, two as moderate, and eight as having a low level. Fourteen biomarkers were found to be significantly increased in childhood obesity/overweight (*p* < 0.05): leptin, insulin, α-amylase, tumor necrosis factor α, interleukin 6, vascular endothelial growth factor-A, C-reactive protein, monocyte chemotactic protein-1, resistin, phosphate, nitric oxide, interleukin 1β, uric acid and fetuin-A; and three were found to be significantly decreased (*p* < 0.05): adiponectin, secretory immunoglobulin A, and interleukin-12p70. In conclusion, the present review supported the idea that saliva might be a promising diagnostic tool in early life and that it is a significant source of obesity biomarkers in children.

## 1. Introduction

Childhood obesity is a global public health problem affecting groups from different socio-economic conditions and involving physical and psychological changes [1]. Outstandingly, in 2022, approximately 160 million children/teenagers aged 5–19 years were obese [2]. Globally is predicted that more than half the population worldwide will be living with obesity and be overweight by 2035 [3]. The central point is that obesity among youth is an essential marker for future chronic noncommunicable pathologies, and those who are obese in early life have higher chances of persisting throughout adulthood [4]. Chronic diseases account for poor quality of life, a significant percentage of the annual healthcare expenditures, depression, and mortalities [5]. Terrifically overweight/obese worldwide costs may achieve USD 4.32 trillion/year by 2035 [3].

Biomarkers can be measurable molecules, genes, or characteristics that serve as surrogate endpoints for biological processes [6,7]. In chronic childhood diseases, biomarkers facilitate early detection and monitoring, thereby promoting health and a better quality of life in the future [6,7,8,9]. Omics approaches have facilitated marker identification in the precision medicine era, primarily due to the availability of panels that provide sensitivity and specificity for current and emerging health states. Additionally, the validation of biomarkers is of paramount importance. While analytical validation is characterized by consistent measurements of a biomarker to the unknown actual values (demonstrating its technical performance), clinical validation relies on the relationship between the biomarker and the interest endpoint [10].

There are also two critical concepts regarding biomarker usage in clinical practice: exploratory associations and actionable thresholds. Exploratory researchers commonly rely on understanding the multifaceted pathways that shape the development of obesity. Actionable information is that which establishes a causal link between noticeable features and the disease’s behavior, justifying new leads and hypothesis investigations [11]. In this context, actionability should be a desirable characteristic of biomarker data, predicting relationships between causal information and therapies/treatments, taking into account broad knowledge systems, technological tools, organizational procedures, key strategic alliances, subject matter experts, financial investments, regulatory and policy structures, and computational algorithms [11].

Regarding biofluids, blood samples are commonly the most widely used marker source [9]. A recent review investigating the biomarkers evaluated during interventions for pediatric obesity noted that 67% of the research assessed estimated biomarkers in whole blood, 40% in plasma, 56% in serum, and 2% in urine [9]. Most obesity-related compounds in the blood are also in saliva, i.e., insulin, leptin, α-amylase, tumor necrosis factor α/interleukin 6, C-reactive protein, and adiponectin. Notably, Goodson’s study compared saliva with plasma insulin in 53 American children (20 obese, 8 overweight, and 25 eutrophic) and found that the salivary concentration highly correlates with plasma levels despite being 50% lower [12]. Another investigation involving Kuwaiti children also found significant correlations between serum and saliva insulin levels, as well as C-reactive protein and adiponectin, with lower values in saliva [13]. Since blood collection is invasive and needle fear is frequent in infants, toddlers, and children, alternative non-invasive and stress-free biofluids are promising.

Saliva is a complex, precise, slightly acidic biological fluid (pH = 6.0–7.0) that contains a mixture of secretions from multiple salivary glands, including the parotid, submandibular, sublingual, and other minor glands below the oral mucosa, as well as liquid from the gingival cleft. Saliva performs multiple physiological functions, including digestion, swallowing and tasting food, lubricating the oral tissues, maintaining tooth integrity, and protecting against bacterial and viral infections [14].

As evidenced by papers in the scientific literature, not many clinician-scientists are focused on children’s biomarkers [6,9] compared to the adult population. Support and training programs for pediatric clinical research are limited, together with the industry’s interest, as they account for a small portion of the market share [6]. Moreover, concerning salivary elements in particular, minor and contradictory data preclude their use as a significant analytical tool in the diagnosis of childhood obesity [6,9]. Insights about the quality/credibility of the available studies and a systematic appraisal of all available results are lacking. Considering that this information is crucial for early diagnosis and assertive intervention aimed at disease control, the present scoping review aims to evaluate the biomarkers in the saliva of children related to obesity. Based on the current quality of the literature regarding the relationship between weight gain and salivary biomarkers, the following question was considered: “Are salivary biomarkers significant factors/indicators for obesity?”

## 2. Materials and Methods

### The Literature Search

**Figure 1:**
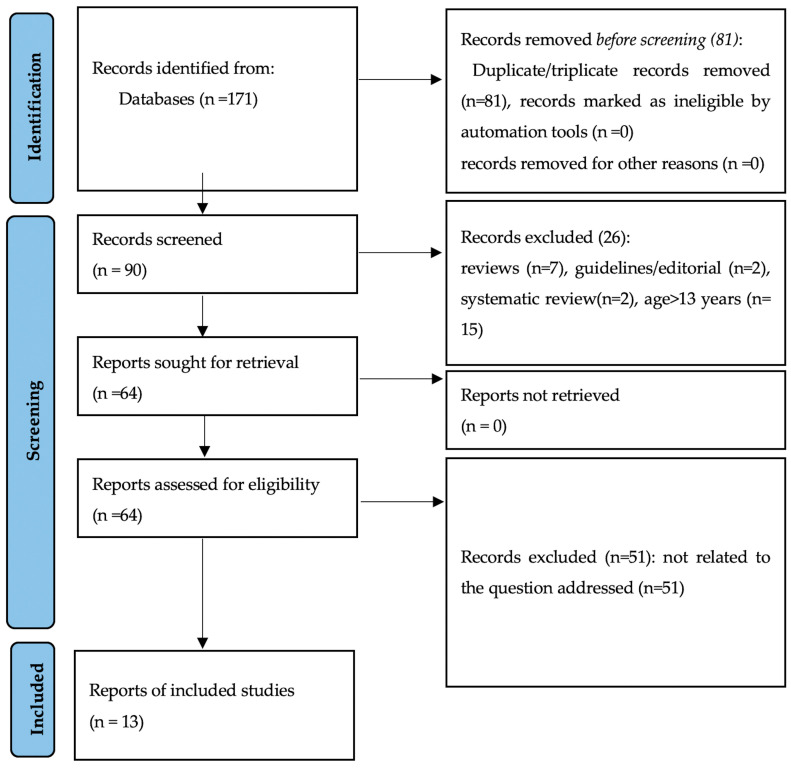
Flow diagram of the literature search in the present review.

The electronic search used the PubMed, Scielo, Scopus, and Embase databases. Papers published from January 1999 to March 2025 were selected. No manual search was performed. Based on the aim of the present scoping review, the following search descriptors (MESH-terms) were used: “obesity and salivary biomarkers and children.” The Preferred Reporting Items for Systematic Reviews and Meta-Analyses (PRISMA) guidelines were followed in the screening process (Figure 1). To be included, the primary outcome should be obesity or overweight based on BMI, children must be younger than 13 at baseline, and the study should have explored the interrelationship with salivary biomarkers. The search was limited to clinical and observational cross-sectional, case–control, or longitudinal studies. Studies concerning adulthood, older people, children, and adolescents with special needs were excluded. Papers written in a non-English language (universal language), letters, guidelines, and literature reviews were dismissed. Papers were not obtained from the grey literature because the reliability and precision of the information can differ significantly, as it is not peer-reviewed and may not have been reviewed or edited.

Assessed articles were carefully classified according to the predetermined criteria Newcastle–Ottawa Scale (NOS) [15] for non-randomized controlled studies (Supplementary Table S1) by two reviewers, F.M.S. and T.M.P., working independently. The disagreement was resolved through further discussion until a consensus was reached. The criteria were also used to assess bias. NOS for cohort/case–control/ evaluates investigations across three key areas: (1) selection of participants, (2) comparability between study groups, and (3) assessment of outcomes/exposure. Within the selection and outcome/exposure categories, there are 4 and 3 criteria, respectively, each worth up to one point. The comparability category has a single criterion that can earn up to two points. The highest score considered was nine points, indicating the lowest risk of bias. For cross-sectional studies, an adaptation was made, excluding two categories in area 1 (selection of controls and definition of controls) and one in area 3 (same method of ascertainment for cases and controls), which received the highest score of 6 points. Studies will be classified as high quality if they achieve a minimum of 3 points in the selection domain, 1 point in comparability, and 2 points in the outcome domain (6 points or more). Those scoring 2 points in selection, at least 1 point in comparability, and 2 points in outcomes will be considered of moderate (fair) quality (minimum of 5 points). Studies will be deemed low quality if they score 0 or 1 point in the selection domain, receive no points for comparability, or score 0 or 1 point in the outcome domain (minimum of 1 point).

## 3. Results

The electronic literature search resulted in 171 articles, of which 81 were duplicates or triplicates. Of the remaining 90 papers, 26 were excluded because the sample of children was older than 13 years at the baseline or because they were not original papers. Fifty-one were also removed because they were not related to the question addressed. Thus, 13 studies were included for critical appraisal and used as the basis for conclusions according to the NOS scale (Supplementary Table S1).

The studies by Goodson et al. [12], Hartman et al. [16], and Shi et al. [17] were classified as high quality because they: 1. selected a representative sample of the exposed/non-exposed cohort, using ascertainment of exposure; 2. compare of the cohorts based on the design/analysis; and 3. consider adequate outcome assessment and a follow-up long enough for outcomes to occur. Despite Alqaderi et al. [13] employing a subsample of the same Kuwait cohort as the three studies above, their subsample was not randomized and not representative, resulting in a total of 5 points and a classification of moderate quality. The same happened with the investigation by Riis et al. [18], which also used a non-randomized subsample of the USA cohort (Table 1).

The seven cross-sectional studies included Starzak et al. [19], Selvaraju et al. [20,21], Naidoo et al. [22], Leme et al. [23], Tvarijonaviciute et al. [24], Hartman et al. [25] and the case–control study of Vitale et al. [26] were rated as having low-quality evidence. This occurred due to the lack of representativeness of the cases, considering the convenience samples used in the methodology for selecting children, as well as the inclusion and exclusion criteria that were not described in detail. Moreover, there was no designation of the non-response rate (Table 1).

**Table 1:** Criteria for paper classification according to the Newcastle–Ottawa Scale (NOS).

*First Author*	*Selection*	*Comparability*	*Outcome/Exposure*	*Design*	*Quality*
Goodson [12]	***	*	**	Random selection from a cohort	High quality
Hartman [16]	***	*	**	Random selection from a cohort	High quality
Shi [17]	***	*	**	Random selection from a cohort	High quality
Alqaderi [13]	**	*	**	Subsample of a cohort	Moderate
Riis [18]	**	*	**	Subsample of a cohort	Moderate
Vitale [26]	*	*	**	Case–control	Low quality
Naidoo [22]	*	*	**	Cross-sectional	Low quality
Hartman [25]		*	*	Cross-sectional	Low quality
Starzak [19]	*	*	**	Cross-sectional	Low quality
Tvarijonaviciute [24]	*	*	**	Cross-sectional	Low quality
Selvaraju [20]	*	*	**	Cross-sectional	Low quality
Selvaraju [21]	*	*	**	Cross-sectional	Low quality
Leme [23]	*	*	**	Cross-sectional	Low quality

Each Astheristic “*” means one point according to the NOS criteria. NOS was adapted to cross-sectional designs, which might have affected the overall methodological quality scores by producing relatively lower values.

**Figure 2:**
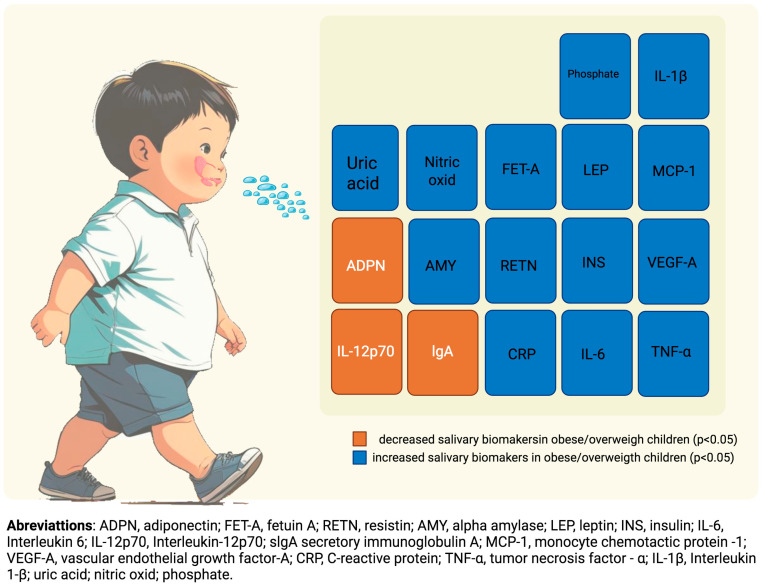
Statistically significant salivary biomarkers identified in the appraised studies.

More than 30 salivary biomarkers were assessed in the thirteen studies appraised, and three were rated as high-quality evidence. Fourteen biomarkers were found to be statistically significantly increased in childhood obesity/overweight (*p* < 0.05): leptin, insulin, α-amylase (AA), tumor necrosis factor α (TNF-α), interleukin 6 (IL-6), vascular endothelial growth factor-A (VEGF-A), C-reactive protein (CRP), monocyte chemotactic protein-1 (MCP-1), resistin, fetuin-A, nitric oxide, interleukin 1β (IL-1β), uric acid, phosphate; and three were found to be statistically significant decreased (*p* < 0.05): adiponectin, secretory immunoglobulin A (IgA), and interleukin-12p70. The summary of the results, including, e.g., *p*-values and confidence intervals, is displayed in Table 2 and Figure 2.

## 4. Discussion

To the best of our knowledge, this review is the first to systematically explore and critically assess the published scientific articles concerning the relationship between salivary biomarkers and obesity/overweight in the pediatric population. A better understanding of this issue reinforced the notion that saliva could be a reliable source of markers associated with childhood obesity and overweight.

In this context, significant biomarkers associated with obesity have been identified in various studies. Special attention is deserved for insulin and CRP, which were higher in children with weight excess in six investigations, and adiponectin, which was diminished in four studies, the majority of which had high quality (Table 1 and Table 2). Furthermore, regarding the interplay between saliva and blood samples (the gold-standard fluid), one included study, rated as high, significantly predicted that 6.4 pmoles/L (128 pg/mL) of salivary insulin would be approximately 67 pmoles/L of plasma insulin [17]. Similarly, a moderate-rated study demonstrated a modest positive correlation between serum and salivary insulin levels [13], and a low negative correlation between adiponectin levels. A strong positive correlation between CRP levels in these two body fluids was found in one more paper, but with a low level of evidence [24].

Biomarkers are quantifiable characteristics of biological processes or any substance that can be measured in the body and influence or predict a disease [27]. In a clinical context, evaluating biomarkers enables professionals to make more informed clinical decisions, helps patients understand their disease, and changes deleterious habits [28] thereby avoiding complications in future health status. Childhood is a critical stage marked by significant changes, providing opportunities for targeted health promotion interventions.

CRP is an acute-phase protein. Fat tissue releases large amounts of inflammatory cytokines, which trigger the liver to produce CRP. Its concentration in the blood plasma increases under inflammatory conditions, particularly as a response to IL-6 release. CRP can bind to components expressed on the surface of apoptotic and damaged cells (lysophosphatidylcholine), activating phagocytosis. CRP has been considered a significant factor linked to obesity in two papers with a high level of evidence [12,17] and with weight excess in one paper with a moderate [13] and three with a low level [20,22,24]. Selvaraju et al. [20] and Goodson et al. [12] showed that salivary CRP was approximately 6-fold higher (median) in overweight/obese children than in eutrophic kids. It is a non-invasive biomarker with good diagnostic value for detecting factors favoring dysregulated metabolism, a common feature in childhood obesity [20]. At room temperature, CRP is stable (≈eight hours), simplifying the saliva collection and making it possible to perform at school or home [29]. In the adult population, salivary CRP is an excellent discriminative measure for relevant CRP serum cut-off points [29], and it’s still debated whether elevated CRP levels are a result of disease or if they play a direct role in the development of chronic conditions.

The association between CRP and childhood obesity/overweight could be supported because obesity is characterized by chronic inflammation at a low-grade level, and CRP is an acute-phase inflammatory compound. Specifically, high CRP concentrations suggest that excess body weight may contribute to a state of chronic low-grade inflammation in children, potentially increasing the risk of developing various future health issues, such as cardiovascular disease and diabetes, through several pathways (e.g., reduced insulin sensitivity, increased release of adhesion molecules by the blood vessel lining, and higher production of clotting factors like fibrinogen in the liver) [30]. In a comprehensive study involving a representative sample of U.S. children (5305—3rd National Health and Nutrition Examination Survey) [31], the proportion of children with CRP blood levels exceeding 2.1 mg/L increased in tandem with higher body mass index (BMI) and no significant associations were observed between CRP levels and age, sex, race, or pubertal stage. In the present review, the investigation of Alqaderi [13], in a subsample with a broad social class/ethnic group of a Kuwait children’s cohort, evaluated salivary CRP analyte at three time points and serum CRP at one time point. This way, the mean (SD) CRP pg/mL in the saliva in children aged 9–11 (visit 1), 11–13 (visit 2), and ≈17 years (visit 3) were very similar, being, respectively: 2.22 (0.71), 2.57 (0.52) and 2.56 (0.56). During the last visit, serum CRP was also estimated, which was twice the salivary values: 5.99 (0.74) pg/mL. In addition, their multiple logistic regression analysis revealed that higher CRP levels were associated with 4.53 more chances of being obese.

Regarding insulin, adiponectin, and leptin, the first hormone is produced in the pancreas, and the others are made in white adipose tissue by mature adipocytes (subcutaneous, visceral, and bone marrow fat). It is well known that insulin is responsible for glucose absorption from the blood into body cells and that adiponectin is involved in regulating glucose levels and facilitating fatty acid breakdown. Leptin influences appetite, satiety, and motivated behaviors toward maintaining energy reserves [32]. The association between increased salivary insulin levels and obesity or overweight in children was found in three studies rated as having high-quality evidence [12,16,17] and three with a low-moderate level [13,21,24]. Irrespective of age, disturbances in glucose metabolism are one of the earliest obstacles encountered in obesity-related metabolic damage. Even though the connection between insulin resistance and obesity is multifaceted and associated with many molecular processes, high insulin levels are commonly associated with insulin resistance in children and adolescents. This circumstance is a precondition to future metabolic problems and diseases, such as diabetes type II and metabolic syndrome [33]. Therefore, the early identification of this marker would undoubtedly be helpful in clinical practice [34]. Higher levels of salivary leptin were associated with obesity in 10- to 12-year-old Kuwaitis in one paper rated as having a high value [12] when waist circumference was considered. Obese individuals stated by abdominal fat demonstrate a superior mean level of circulating blood leptin, leading to significant amounts in saliva, highlighting the close connection between this adipokine and the fat tissue [12]. When saliva was compared to serum levels in youth, the leptin detectability rate in saliva was circa 20% lower [13].

On the other hand, decreased salivary levels of adiponectin (anti-inflammatory adipokine) were linked to excess weight in childhood in two papers of high quality [12,17] and two rated as moderate-low level of evidence [13,21]. In the study of Goodson et al., [12] salivary adiponectin diminished by approximately 30% with growing obesity in lean children [12]. Even when obese children were compared with overweight ones, a significant decrease could be identified [21]. In a predictive analysis for obesity, adiponectin reached significance (OR 0.54 [95%CI: 0.30, 0.90]; *p* = 0.044) [13] and showed equivalent detectability rates in saliva and serum (98.6%). Adiponectin was one of the top-ranking factors identified by three algorithms in the logistic regression models when the body mass index was employed for obesity classification [17].

Regarding AA and IgA, only one study designated as low quality investigated the enzyme and the antibody and revealed that obese children had significantly higher levels of AA in the saliva than overweight and eutrophic ones and substantially lower IgA rate compared to regular weight [19]. Their multiple linear regression models indicate that BMI can predict AA and IgA secretion rates in 10-year-old South African children [19]. The low-grade systemic inflammation associated with obesity may chronically activate the stress response system [35]. Salivary glands produce AA under sympathetic stimulation. This enzyme is predominant in saliva and positively connected with β-blockers, indicating stress-associated autonomic nervous system activity [36]. IgA is one of the most abundant immunoglobulins in the human body, with a peak concentration typically occurring around 7 years of age. It acts as the first line of defense against antigens and microbial colonization, playing a crucial role in mucosal homeostasis in the mouth, stomach, intestine, respiratory tract, and genitourinary tract. It has been proposed that IgA is also important in immunoregulation [37]. In this context, obesity may be linked to body alertness and poorer mucosal immunity conditions. 

TNF-α is a potent activator of cellular apoptosis, and the adipose tissue can produce high amounts of this cytokine, both from adipocytes and macrophages infiltrated in the adipose tissue. TNF-α stimulates the activation of the nuclear factor kappa-B (NF-kB), a transcription factor responsible for regulating inflammation-related genes [38]. Thus, in obesity, TNF-α increases adipokine production, mediated by enhanced NF-κB pathway activity [38]. Animal trial suggested that deleting TNF-α protects from the obesity-related decrease in insulin receptor signaling in fat tissues and muscles [39], being a central factor contributing to insulin resistance in diet-induced obesity. It is a key regulator of IL-6, which is also produced in adipocytes and macrophages. Many physiological and pathological factors influence IL-6 secretion, including stress, dietary habits, hormones, cytokines, diet, and physical activity [40]. IL-6 derived from the adipose tissue impacts the metabolism through lipoprotein lipase downregulation, insulin sensitivity, adipose tissue-specific gene expression, and triglyceride release, for example [41]. In the present review, two studies rated as having a low value of evidence found an association between obesity and higher amounts of salivary TNF-α, one involving Brazilian preschoolers with dental caries [23] and the other comprising North American children aged 6 to 10 years [20]. The latter study [20] also demonstrates that IL-6 is a biomarker significantly higher in overweight/obese children compared to eutrophic individuals. A previous systematic review corroborates our findings regarding TNF-α but not IL-6 [40]. Perhaps TNF-α could be a more potent marker, better reflecting the chronic inflammation associated with obesity, and deserving of further investigation. Of interest, a combination of cytokines, including IL-6 and TNFα, has already been reported to induce hepatocyte nitric oxide synthase expression [42]. Nitric oxide is a potent biological mediator and a peripheral inflammatory biomarker. Vitale’s study [26], included in our review, demonstrated that its concentration in saliva was increased in children with obesity and overweight.

IL-12p70 is part of a cytokine group that plays a role in the immune system. This heterodimer comprises subunits (p40 and p35) and is essential in activating T cells (e.g., T helper, natural killer/gamma-delta T cells), showing anti-angiogenic properties [43]. On the other hand, VEGF-A is a dimeric glycoprotein that plays a crucial role in angiogenesis [44]. The investigation of Hartman et al. [16] of a high level of evidence considered a random selection of adolescents from a Kuwait cohort and showed that elevated salivary glucose was associated with elevated VEGF-A and reduced IL-12p70 in the saliva in the obesity condition. They have supported the hypothesis that developing a bigger body mass might be connected to increased vascularization to supply this extra tissue mass and, consequently, increased VEGF-A and reduced amounts of IL-12p70. Although this vascular adaptation may be beneficial, the formation of irregular vessels could be a contributing factor to the aggravation of cardiovascular diseases [45]. A statistically significant correlation between salivary IL-1β and obesity was found in Spanish children [24]. The overexpression of IL-1 in adipose tissues causes immune cell infiltration, resulting in low-grade inflammation [46], which happens in obesity. 

In the present review, the last two investigated cytokines that reached statistical significance were MCP-1 and resistin, which were increased in overweight or obese children [20]. MCP-1 regulates the migration and infiltration of monocytes/macrophages from the bloodstream across the vascular endothelium to the sites of inflammation or for routine immunological surveillance of the body tissues [47]. Resistin is chiefly secreted by macrophages and is coupled with cardiovascular diseases [48,49] probably because it promotes inflammation, dysfunction of vascular endothelial cells, and apoptosis in smooth muscle cells [48]. Due to the subclinical inflammation condition (characteristic of obesity), the higher MCP-1 and resistin make proper sense. Moreover, the accumulation of monocytes in blood vessels, favored by these cytokines, may lead to the formation of atherosclerotic plaques, reinforcing the notion that obesity is a significant risk factor for atherosclerotic heart disease [50].

One of the most recent investigations, despite low quality, suggested that salivary fetuin-A was significantly enhanced in overweight/obese kids. This could be an excellent diagnostic biomarker, as, according to the area under the ROC curve (80% of the time), there is a probability of diagnosing children (with or without the disease) [21]. Fetuin-A is a glycoprotein secreted by the adipose tissue and the liver and is linked to reduced insulin sensitivity and glucose tolerance, impairing glycemic control. It is commonly raised in obesity as well as metabolic syndrome, diabetes mellitus (type 2), nonalcoholic fatty liver disease, and vascular calcification in individuals with obese chronic kidney disease [51].

Salivary levels of phosphate ions were found to be high in obese/overweight North American children. They may be connected to different functions of phosphate-regulating machinery from salivary glands, possibly related to fat cell turnover, as reported by one study appraised in the present review [25]. This study did not find the same association in the blood serum. Therefore, it was supposed that salivary phosphate might be an early biomarker of metabolic disturbance in predicting obesity. Considering that poor evidence was obtained for the above investigation, further research should target the mechanisms involved in phosphate accumulation in children with excess weight to sustain this supposition. Recently, salivary uric acid has been reported as a marker for obesity in American children [18] in a fair-quality study correlating general BMI and this variant. The literature demonstrates a strong correlation between serum and saliva [52,53], but the exact mechanisms underlying obesity and hyperuricemia are not well understood. Still, it could be supposed that increased adiposity favors overall nucleic acid metabolism, raising uric acid synthesis through purine breakdown [54]. In addition, changes in glomerular hemodynamics due to obesity may superactivate the renin–angiotensin–aldosterone system and lead to nephropathy, thereby diminishing uric acid excretion [54]. Additionally, uric acid may influence the type 2 inflammatory response and vascular restriction in the cardiovascular system [55].

Childhood obesity is a worrying theme, considering its high prevalence around the globe and the relevant psychological and physiological effects [1,2]. Thus, new insights in this respect offer significant benefits. Although papers appraised in the present review emphasize the role of salivary biomarkers in understanding childhood obesity and its associated metabolic risks, this should be interpreted cautiously. Firstly, the heterogeneity among the studies precluded a meta-analysis. Then, 30% of the studies were conducted with the Kuwaiti population [12,13,16,17], with a subsample of the same cohort. Kuwait is the 21st country with childhood obesity [56], and cohort studies are often time-consuming and expensive, which is why this type of study is being explored. The actual readiness of salivary biomarkers for clinical implementation and the establishment of trusty reference values for saliva in clinical practice face several barriers, including regulatory and ethical aspects associated with salivary diagnostics, standardization methods for analysis, storage, collection, and correlation with plasma levels, taking into account population characteristics. Other limitations regarding saliva as a diagnostic fluid, including variability in flow rate, sample contamination, intra-individual and diurnal variation, fasting state, and storage stability [57], should also be pondered. All of these factors influenced the reproducibility and accuracy of the clinical routine practice of an ideal biomarker. Regarding the weaknesses mentioned above, translating salivary analyte levels into clinically meaningful thresholds is challenging, and the relationship between saliva and serum concentrations warrants further exploration.

Preventing obesity in childhood is especially critical, as the harmful metabolic and inflammatory changes can continue over time and lead to serious health problems and comorbidities in adulthood. Even with the fragilities stated above, investigating salivary biomarkers should be encouraged for the early prediction of obesity, thereby facilitating effective and immediate intervention in infancy to control obesity-related diseases. In the era of precision medicine, tests involving biomarkers in pediatrics, which favor early intervention and disease control, are crucial for population health. 

## 5. Conclusions

In conclusion, this scoping review supports the hypothesis that saliva may be a promising diagnostic tool in early life and a significant source of obesity biomarkers in children, since a standardized collection process has been established, followed by validation and integration into existing clinical pathways. Despite saliva’s potential, the discovery of biomarkers in this fluid is limited in the pediatric population and warrants further exploration.

## Figures and Tables

**Table 2:** Characteristics of the included studies.

First Author/Year	Country	Subjects	Age (Years)	Saliva Analysis	Analyzed Markers	Statistically Significant Association Between Salivary Markers in Eutrophic Children and:		
						Obesity	Overweight	Obesity/Overweight
Naidoo 2012 [22]	Africa	170	9.41 ± 1.55	ELISA	CRP			↑ CRP *[6.77(0.92) × 7.31(0.93)* *p < 0.05] ^a^*
Hartman 2013 [25]	USA	77	10.5 ± 1.8	Chromatography/mass spectrometry	Phosphate			↑ Phosphate *[≈0.9(0.35–1.2) × 1.25(0.45–2.2)* *p < 0.05] ^b^*
Goodson 2014 [12]	USA Kuwait	53 + 744	10–12	Luminex System	insulin, IFN-γ, IL-10, IL-12p70, IL-13, IL-17A, IL-1β, IL-4, IL-6, IL-8, MCP-1, TNF-α, VEGF-A, ghrelin, leptin, MMP-9, adiponectin, CRP, resistin	↑ CRP *[73.01(153.75)/77.15(186.95) × 429.44(668.52)/443.13(1033.29)* *p < 0.0001] ^c^* ↑ Insulin *[39.39(45.38)/44.70(54.38)* × *112.98(125.09)/143.50(150.24)* *p < 0.0001] ^c^* ↑ Leptin *[1.06(4.77)/0.63(4.61) × 3.16(6.40)/3.70(6.41)* *p < 0.0001] ^c^* ↓ Adiponectin *[4220(5303)/3994(5052) × 2548(2779)/3062(3752)* *p < 0.0001] ^c^*	↑ CRP *[73.01(153.75)/77.15(186.95) × 177.46(311.93)/281.39(516.54)* *p < 0.0001] ^c^* ↑ Insulin *[39.39(45.38)/44.70(54.38) × 80.39(88.74)/76.25(87.13)* *p < 0.0001] ^c^* ↓ Adiponectin *[4220(5303)/3994(5052)x 2402(3785)/3322(3693)* *p = 0.0001] ^c^*	
Vitale 2014 [26]	Italy	45	9.4 ± 0.6	ELISA	Nitric Oxide			↑ Nitric Oxide *[≈226 × 283]* *p < 0.0001] ^d^*
Starzak 2016 [19]	Africa	132	10.05 ± 1.68	ELISA	Alpha amylase, IgA	↑ Alpha amylase *[79.83(43.12)/62.13(36.06) × 122.75(46.50)* *p < 0.001] ^e^* ↓ IgA *[243.95(119.23) × 158.34(56.03)* *p < 0.001] ^e^*	↓ IgA *[B = −45.737(10.09)* *p < 0.0001] ^e^*	
Hartman 2016 [16]	Kuwait	744	10 ± 0.7	Luminex System	insulin, IFN-γ, IL-10, IL-12p70, IL-13, IL-17A, IL-1β, IL-4, IL-6, IL-8, MCP-1, TNF-α, VEGF-A, ghrelin, leptin, MMP-9, adiponectin, CRP, resistin	↑ VEGF-A *[0.10(0.04)* *p < 0.01] ^f^* ↑ Insulin *[0.15(0.04)* *p < 0.0001] ^f^* ↓ IL-12p70 *[−0.14(0.05)* *p < 0.0001] ^f^*	↑ Insulin *[0.25(0.11)* *p < 0.02] ^f^*	
Tvarijonaviciute 2019 [24]	Spain	129	8–12	Luminex System	Glucose, triglycerides, IL-1β, IL-6, IL-8, insulin, leptin, MCP-1, NGF, HGF, TNF-α, CRP			↑ Insulin *[6.41(3.8–19) × 17(5.94–62)* *p = 0.003] ^g^* ↑ CRP *[2.04(0.51–7.01) × 5.79(2.37–12)* *p ≤ 0.001] ^g^* ↑IL-1β *[6.52(2.33–23) × 17(8.32–36)* *p ≤ 0.028] ^g^*
Shi 2019 [17]	Kuwait	744	10 ± 0.67	Luminex System	insulin, C-reactive protein (CRP), adiponectin, leptin, IL-1β, IL-4, IL-6, IL-8, IL-10, IL-12P70, IL-13, IL-17A, resistin, MMP-9, MPO, MCP-1, TNF-α, VEGF-A, IFN-C, ghrelin, IL-17A, IFN-γ	↑ CRP ↑ Insulin ↓ Adiponectin *[AUC 0.820 (0.782–0.862)] ^h^*		
Selvaraju 2019 [20]	USA	76	6–10	Luminex System	CRP, resistin, MCP-1, TNF-α, IL-6, complement factor D, IL-10			↑ CRP *[AUC 0.866(0.780–0.952)* *p = 0.0001] ^h^* ↑ IL-6 *[AUC 0.673(0.554–0.801)* *p = 0.01] ^h^* ↑ MCP-1 *[AUC 0.715(0.554–0.801)* *p = 0.002] ^h^* ↑ Resistin *[AUC 0.731(0.606–0.855)* *p = 0.001] ^h^* ↑ TNF-α *[AUC 0.694(0.564–0.825)* *p = 0.005] ^h^*
Selvaraju 2022 [21]	USA	76	6–10	Luminex System	Fetuin A, insulin, adiponectin	↑ Fetuin A *[≈50 × 400 p < 0.01] ^i^* ↑ Insulin *[≈100 × 400 p < 0.001] ^i^* ↓ Adiponectin *[≈25 × 8 p < 0.003] ^i^*	↑ Fetuin A *[≈50 × 407 p < 0.004] ^i^*	
Leme 2022 [23]	Brazil	94	4–5	Luminex System	TNF-α	↑ TNF-α *[1.13(1.09–1.61) p < 0.001] ^j^*		
Alqaderi 2022 [13]	Kuwait	353	10–17	Luminex System	Insulin, CRP, adiponectin, leptin, IL-6, IL-8, IL-10, MCP-1, VEGF	↑ CRP *[4.53(2.4–8.50) p ≤ 0.001] ^k^* ↑ Insulin *[3.29(1.82–5.97) p ≤ 0.001] ^k^* ↓ Adiponectin *[0.54(0.3–0.9) p ≤ 0.044] ^k^*		
Riis 2023 [18]	USA	217	0–12	Colorimetric enzymatic assay	Uric acid			↑ Uric acid *[0.13/0.17 p ≤ 0.01/p < 0.0001] ^l^*

Values refer to: a = mean (SD) pg/mL; b = med (min–max) mg/dL; c = male med (IQR)/female med (IQR) pg/mL; d = mean µmol/L; e = mean (SD) U/mL, µg/mL; f = estimates (SE); g = med (Q25–Q75%); pg/mL, ng/mL; h = area under curve (95% CI); i = mean ng/mL; j = rate ratio (95% CI); k = odds ratio (95% CI); l = correlation coefficient.

## Supplementary Materials

The following supporting information can be downloaded at: https://www.mdpi.com/article/10.3390/ijms26125789/s1, Table S1: The Newcastle–Ottawa Scale, a Quality Assessment.
