# Supplementary material for: Exploring Salivary Biomarkers in Pediatric Obesity: A Scoping Review
Source: Int J Mol Sci. 2025 Jun 17;26(12):5789. doi: 10.3390/ijms26125789 (PMC12193587; doi:10.3390/ijms26125789)
Supplement: Supplementary file 1 [file ijms-26-05789-s001.zip › ijms-3639134-supplementary.pdf]

Supplementary Table 1. The Newcastle–Ottawa Scale, a Quality Assessment.

CASE CONTROL STUDIES	COHORT STUDIES	CROSS-SECTIONAL STUDIES (ADAPTATION)
	**Selection (area 1)**	
1) <u>Is the case definition adequate</u>?<br>a) yes, with independent validation *<br>b) yes, e.g., record linkage or based on self-reports<br>c) no description<br>2) <u>Representativeness of the cases</u><br>a) consecutive or obviously representative series of cases *<br>b) potential for selection biases or not stated<br>3) <u>Selection of Controls</u><br>a) community controls *<br>b) hospital controls<br>c) no description<br>4) <u>Definition of Controls</u><br>a) no history of disease (endpoint) *<br>b) no description of source	1) <u>Representativeness of the exposed cohort</u><br>a) truly representative of the average __________ in the community *<br>b) somewhat representative of the average _________ in the community *<br>c) selected group of users e.g., nurses, volunteers<br>d) no description of the derivation of the cohort<br>2) <u>Selection of the non exposed cohort</u><br>a) drawn from the same community as the exposed cohort *<br>b) drawn from a different source<br>c) no description of the derivation of the non-exposed cohort<br>3) <u>Ascertainment of exposure</u><br>a) secure record (e.g., surgical records) *<br>b) structured interview *<br>c) written self-report<br>d) no description<br>4) <u>Demonstration that outcome of interest was not present at start of study</u><br>a) yes *<br>b) no	1) <u>Is the case definition adequate</u>?<br>a) yes, with independent validation *<br>b) yes, e.g., record linkage or based on self-reports<br>c) no description<br>2) <u>Representativeness of the cases</u><br>a) consecutive or obviously representative series of cases *<br>b) potential for selection biases or not stated
	**Comparability (area 2)**	
1) Comparability of cases and controls on the basis of the design or analysis<br>a) study controls for _________ *<br>b) study controls for any additional factor *	1) <u>Comparability of cohorts on the basis of the design or analysis</u><br>a) study controls for ________*<br>b) study controls for any additional factor *	1) Comparability of cases and controls on the basis of the design or analysis<br>a) study controls for __________ *
	**Exposure/Outcome (area 3)**	
1) <u>Ascertainment of exposure</u><br>a) secure record *<br>b) structured interview where blind to case/control status *<br>c) interview not blinded to	1) <u>Assessment of outcome</u><br>a) independent blind assessment *<br>b) record linkage *<br>c) self-report<br>d) no description	1) <u>Ascertainment of exposure</u><br>a) secure record *<br>b) structured interview where blind to case/control status *<br>c) interview not blinded to

case/control status

d) written self-report or medical record only

e) no description

2) <u>Same method of ascertainment for cases and controls</u>

a) yes *

b) no

3) <u>Non-Response rate</u>

a) same rate for both groups *

b) non-respondents described

c) rate different and no designation

2) <u>Was follow-up long enough for outcomes to occur</u>

a) yes (select an adequate follow up period for outcome of interest) *

b) no

3) <u>Adequacy of follow up of cohorts</u>

a) complete follow up - all subjects accounted for *

b) subjects lost to follow up unlikely to introduce bias - small number lost - > ____ % (select an adequate %) follow up, or description provided of those lost) *

c) follow up rate < ____% (select an adequate %) and no description of those lost

d) no statement

case/control status

d) written self-report or medical record only

e) no description

2) <u>Non-Response rate</u>

a) same rate for both groups *

b) non-respondents described

c) rate different and no designation

Based in: ***https://www.ohri.ca/programs/clinical_epidemiology/oxford.asp***
